# Lady Health Inspectors in Kensington

**Published:** 1904-03-05

**Authors:** 


### Lady Health Inspectors in Kensington.

We have on more than one occasion insisted upon the advantages of educating the masses rather than trying to force them by too hasty legislation into the practice of proper habits of hygiene. Among the many methods by which instruction may be conveyed into the quarters where it is most needed, not the least valuable is the employment of women as health inspectors. A good example of the success which may be achieved in this way is shown in a recently-issued report by the Medical Officer of Health for Kensington, which includes an account of the work done during 1903 by the two lady inspectors, Miss De Chaumont and Miss Looker. In addition to their duties of inspecting workshops where women are employed, these ladies were authorised to carry out investigations with respect to phthisis and deaths from infantile diarrhœa. In 1901 the Kensington Borough Council resolved to make pulmonary tuberculosis voluntarily notifiable by medical practitioners, and in order to utilise the information thus obtained it was decided to employ the lady inspectors to inquire into the circumstances of the cases notified, and to give suitable instruction to the patient or the relatives concerning the hygienic principles to be observed in the patient's home. During the past year 221 cases were notified in the district, and an inquiry was made into every one of these, and also with regard to 26 deaths of persons whose cases had been notified in 1902, and 127 deaths of persons whose illness had not been notified; the total cases investigated being 374. Forty-seven of these patients were residing in common lodging-houses. The inspectors state that in the majority of instances the rooms occupied by the sick persons were found to be fairly clean and ventilated. Generally speaking, a distinct improvement on the previous year was noticed in ventilation by open windows, and increased appreciation of the value of fresh air, as also of the danger arising from indiscriminate spitting. In each case instructions were given to the patient or his relations enjoining the necessity of wet cleansing of rooms, furniture, etc., and with respect to the treatment of sputa.

Even more important than the foregoing was the work carried out with regard to the deaths from infantile diarrhœa. The lady inspectors were enjoined to interview the mothers in order to discover, if possible, to what extent the mortality might be attributed to preventable causes. This somewhat trying duty appears to have been performed with the greatest tact and good judgment. The inspectors report that commonly a doctor was called in too late in the illness to be of service, the delay being sometimes due to the slight importance attributed to the illness at its onset, but also in part explained by the poverty of the parents and their unwillingness to make application for "medical relief."

Particular inquiry was made as to the foods upon which the infants had been fed. As a rule but little fresh cow's milk was given, and it appeared that skimmed milk or condensed milk was commonly used; moreover, the latter was generally prepared from "separated" milk and therefore contained a great deficiency of fat. In the case of the ordinary milk supplied, it is pointed out that it seldom arrives to the consumer till twelve or more hours after the cows are milked, and no adequate measures are taken, as a rule, to keep it cool at the farm or on the railway journey, while it is liable to be fouled with dirt in every stage of its production and progress. Seeing that milk is such an excellent medium for the growth of pathological micro-organisms, it is small wonder that with such contamination gastro-enteritis is frequently caused. The remedy for the evil, the inspectors believe, is to be sought in improved homes, better training of girls to fit them for their duties as wives and mothers, and in a purer milk supply, such as could be provided by means of "Municipal Milk Depôts," as is now done with good results in Battersea.

The inspectors make the suggestion that it might be possible to utilise "health visitors" to visit every home as soon as practicable after the birth of a child has been registered, to give advice to ignorant mothers with regard to the rearing of their children. It is interesting to note that only two of the 74 children who died of infantile diarrhœa were said to have been breast-fed throughout.

It will appear from the above that the lady health inspectors of Kensington have not only been able to collect much useful information, but have also been instrumental in carrying out an effective little campaign against ignorance and its consequent waste of human life.

